# Gene Expression Profiling of Early Acute Febrile Stage of Dengue Infection and Its Comparative Analysis With *Streptococcus pneumoniae* Infection

**DOI:** 10.3389/fcimb.2021.707905

**Published:** 2021-10-28

**Authors:** Leena H. Bajrai, Sayed S. Sohrab, Thamir A. Alandijany, Mohammad Mobashir, Muddassir Reyaz, Mohammad A. Kamal, Ahmad Firoz, Shabana Parveen, Esam I. Azhar

**Affiliations:** ^1^ Special Infectious Agents Unit – BSL-3, King Fahd Medical Research Centre, King Abdulaziz University, Jeddah, Saudi Arabia; ^2^ Biochemistry Department, Faculty of Sciences, King Abdulaziz University, Jeddah, Saudi Arabia; ^3^ Department of Medical Laboratory Technology, Faculty of Applied Medical Sciences, King Abdulaziz University, Jeddah, Saudi Arabia; ^4^ SciLifeLab, Department of Oncology and Pathology Karolinska Institutet, Stockholm, Sweden; ^5^ Department of Healthcare Management, Jamia Hamdard Hamdard Nagar, New Delhi, India; ^6^ West China School of Nursing/Institutes for Systems Genetics, Frontiers Science Center for Disease-related Molecular Network, West China Hospital, Sichuan University, Chengdu, China; ^7^ King Fahd Medical Research Center, King Abdulaziz University, Jeddah, Saudi Arabia; ^8^ Enzymoics, Novel Global Community Educational Foundation, Hebersham, NSW, Australia; ^9^ Department of Biological Sciences, Faculty of Science, King Abdulaziz University, Jeddah, Kingdom of Saudi Arabia; ^10^ Department of Bioscience, Jamia Millia Islamia, New Delhi, India

**Keywords:** dengue virus, *Streptococcus pneumoniae*, pathogenesis, gene expression, innate immunity, dengue hemorrhagic fever, vascular fragility, comparative genomics

## Abstract

Infectious diseases are the disorders caused by organisms such as bacteria, viruses, fungi, or parasites. Although many of them are permentantly hazardous, a number of them live in and on our bodies and they are normally harmless or even helpful. Under certain circumstances, some organisms may cause diseases and these infectious diseases may be passed directly from person to person or *via* intermediate vectors including insects and other animals. Dengue virus and Streptococcus pneumoniae are the critical and common sources of infectious diseases. So, it is critical to understand the gene expression profiling and their inferred functions in comparison to the normal and virus infected conditions. Here, we have analyzed the gene expression profiling for dengue hemorrhagic fever, dengue fever, and normal human dataset. Similar to it, streptococcus pneumoniae infectious data were analyzed and both the outcomes were compared. Our study leads to the conclusion that the dengue hemorrhagic fever arises in result to potential change in the gene expression pattern, and the inferred functions obviously belong to the immune system, but also there are some additional potential pathways which are critical signaling pathways. In the case of pneumoniae infection, 19 pathways were enriched, almost all these pathways are associated with the immune system and 17 of the enriched pathways were common with dengue infection except platelet activation and antigen processing and presentation. In terms of the comparative study between dengue virus and Streptococcus pneumoniae infection, we conclude that cell adhesion molecules (CAMs), MAPK signaling pathway, natural killer cell mediated cytotoxicity, regulation of actin cytoskeleton, and cytokine-cytokine receptor interaction are commonly enriched in all the three cases of dengue infection and Streptococcus pneumoniae infection, focal adhesion was enriched between classical dengue fever — dengue hemorrhagic fever, dengue hemorrhagic fever—normal samples, and SP, and antigen processing and presentation and Leukocyte transendothelial migration were enriched in classical dengue fever —normal samples, dengue hemorrhagic fever—normal samples, and Streptococcus pneumoniae infection.

## Introduction

Germs or microbes are found everywhere in the air, soil, and water as well as on the bodies of different host species. Many of them are usually harmless and a few of them could be even helpful to minimize the risk of acquiring sickness. However, Germs or microbes can be pathogenic and may lead to devistating outcomes. Indeed, the infectious diseases are among the leading cause of death and are responsible for approximately 14.9 million deaths globally (Morens et al., 2004; Jones et al., 2008; Siemieniuk et al., 2011; Casanova and Abel, 2013; Wu et al., 2020). Infectious diseases are illnesses caused by pathogenic microorganisms such as bacteria, viruses, parasites, or fungi that can be transmitted from one individual to another either directly or indirectly (vector-borne). An infectious disease develops when the immune system of the host is weakened or when the infectious agent overwhelms the immune system (Halstead, 1988; Chen et al., 1997; Ubol et al., 2008; Rothman, 2011; Costa et al., 2013; Datan et al., 2016; Zhu et al., 2020).

Dengue is a viral infection caused by four types of viruses (DENV-1, DENV-2, DENV-3, and DENV-4){Guzman:2016js, Monath:1994cq, Tripathi:2013wu, Pierson:2020ds, Firth:2013bb, Holmes:2003dx, Clarke:2002bp, Bardina:2017dd}. These viruses are transmitted through the bite of infected *A. aegypti* and *A. albopictu*s female mosquitoes that feed both indoors and outdoors during the daytime (from dawn to dusk). The clinical manifestations of dengue range from acute self-limiting febrile illness to life-threatening dengue hemorrhagic fever and the dengue shock syndrome{Ubol:2008eq, Warsi:vd, Halstead:1988iy}. More than 2 billion people in tropical and subtropical regions are at risk of dengue virus infection, leading to 50 to100 million human infections and 24,000 deaths annually. Currently, there are a few antiviral therapies for dengue but they are not highly effective (Eong Ooi and G, 2011; T, 2012; DH, 2013; Firth and Lipkin, 2013). It has been shown that host response to DENV (dengue virus) infection can now be presented in two distinct phases by using unique transcriptional markers in which DHF (dengue hemorrhagic fever) signatures that have been identified during days 1–3 may have applications in developing early intraepidemic evolution of the circulating DENV and might be responsible for increased severity of disease, suggesting that the circulating DENV might have become more virulent through passage in hosts during the epidemic stage (R et al., 1993; Cordeiro et al., 2007; Martina et al., 2009; Morrison et al., 2010). Molecular diagnostics for DHF (Cordeiro et al., 2007; Ubol et al., 2008; Sun et al., 2013; Sim and Hibberd, 2016) have shown the global gene expression patterns (Sun et al., 2013). It has also been shown that some of the gene expression signatures displayed accuracy of more than 95% which indicates that gene expression profiling with these signatures may prove an important step for DHF prognosis at the earlier stages during infection (Nascimento et al., 2009). There are a number of previous works where the gene expression profiling has been studied in DF (classical dengue fever) and DHF and presented promising perspectives. SP (Streptococcus pneumoniae) is also an infectious pathogen. The diseases caused by it are classified as pneumococcal diseases and pneumonia is currently the most common pneumococcal disease. Pneumococcal disease refers to any infection caused by Streptococcus pneumoniae, also known as pneumococcus. Ear and sinus infections, as well as pneumonia and bloodstream infections, are all possible Pneumococcal infections. Pneumococcal disease can be prevented with vaccination. It is a global health issue and affects children under the age of five as well as the elderly and individuals with pre-existing health conditions. In general, SP colonizes the nasopharynx of its host and with the passage of time, migrate to the tissues and organs and lead to infections (van der Poll and Opal, 2009; Siemieniuk et al., 2011; Didelot et al., 2012; Chen and Kolls, 2013; Cubas et al., 2013; Pengo et al., 2013).

Continuing with ongoing issues, we have mainly presented the comparative study of DENV and SP gene expression profiling and its potential impact on the signaling pathways in a simplified way. For this purpose we have not only analyzed the DEGs (differentially expressed genes) and inferred pathways but also analyzed the networks of DEGs. Furthermore, distinct gene expression patterns were found in patients with various etiologies of respiratory infections. As a result, microarray studies of patient peripheral blood leukocytes may help with infectious disease differential diagnosis.

## Results

In this study, we have used the publicly available array expression profiling dataset from GEO (Gene Expression Omnibus) which contains dengue hemorrhagic fever [DHF (9 patients)], dengue fever [DF (9 patients)], and control (normal) sample [ND (8 uninfected with dengue)]. Here, our main goal is to study the role of NS5 in the immune system and associated pathways of dengue virus-infected humans. Furthermore, for pneumoniae, we have used the GSE6269 dataset and performed the expression profiling followed by the pathway enrichment and finally compared DENV and SP at both the expression and functional levels.

### Gene Expression Profiling During Early Acute Febrile Stage of Dengue Infection Can Predict the Disease Outcome

**Figure 1:**
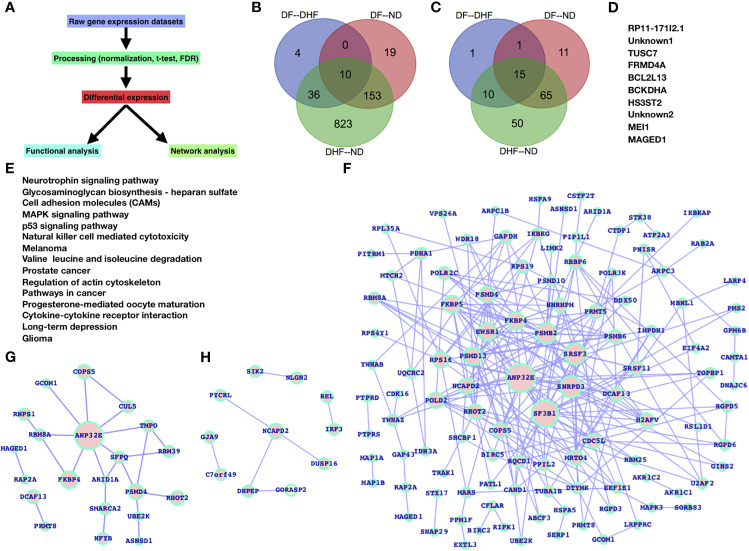
Gene expression profiling in case of dengue infection: **(A)** workflow. Venn diagram for **(B)** DEGs and **(C)** inferred pathways. **(D)** common DEGs between all the samples **(E)** commonly inferred pathways for all the samples. DEG networks **(F)** DHF *versus* ND, **(G)** DF *versus* ND, and **(H)** DF *versus* DHF.

For the selected datasets, the DEGs (differentially expressed genes) and their inferred pathways have been identified in different combinations (DF versus ND, DHF versus ND, and DF versus DHF). We have applied a simplified approach for the entire analysis (**Figure 1A**).

### Dengue Virus Drives Potential Change in Large Number of Genes Expression During Dengue and Dengue Hemorrhagic Fever

Based on our findings, gene expression patterns display significant variation in terms of expression level and after dengue virus infection. A large number of genes had their expression levels changed. Overall gene expression and the number of inferred pathways change from ND to DF and DHF, primarily in DHF (**Figures 1B, C**). There are only 10 genes that are shared across all samples, and there are 15 pathways that are shared across all samples. In comparison to the others, DF-DHF specific genes and pathways are four and inferred pathways are only one, DF-ND specific genes and pathways are 19 and 11, and DHF—ND specific genes and pathways are very high (823 DEGs and 50 pathways) (**Figures 1B, C**). Between DHF and ND, the number of shared DEGs and pathways is relatively high. The total number of shared DEGs and pathways was 10 and 15, respectively (**Figures 1D, E**). RP11-171I2.1, TUSC7, FRMD4A, BCL2L13, BCKDHA, HS3ST2, MEI1, MAGED1, and two unknown genes are among the genes that have been identified. The majority of these common DEGs are linked to human diseases, primarily cancer, and are known to regulate essential biological processes (such as apoptosis) as well as affect potential biological process components. Similarly, the majority of the frequently enriched pathways are grouped together.

We also performed a network study on the DEGs to get a network-level understanding of how these genes changed their expression patterns (**Figures 1F–H**). **Figure 1F** depicts the DHF *versus* ND DEGs network, which is supplemented by DF *versus* ND (**Figure 1G**), and DF *versus* DHF (**Figure 1H**).

### Top-Ranked Genes Infer the Most Critical Signaling Pathways

**Figure 2:**
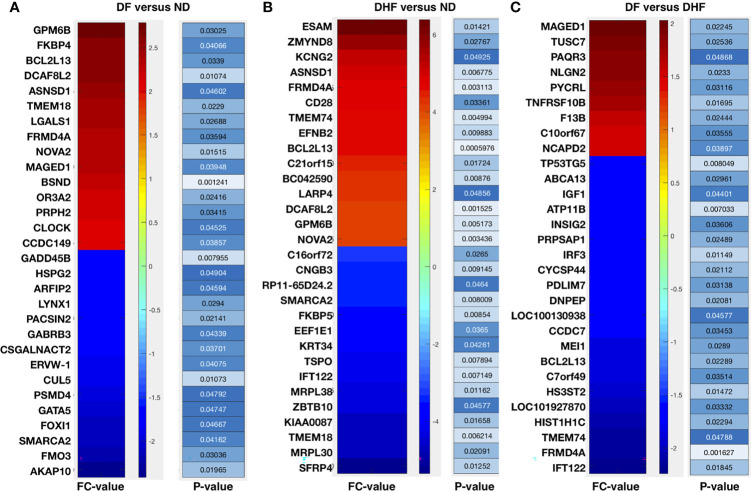
Top 30 DEGs followed by their fold changes and p-values: **(A)** DF *versus* ND **(B)** DHF *versus* ND, and **(C)** DF *versus* DHF.

Here, we have selected the top 30 genes (up (fold change >= +2.0 and p-value <= 0.05) and down (fold change <= -2.0 and p-value <= 0.05) regulated) and analyzed the inferred pathways which leads to the critical pathways such as T-cell activation, Angiogenesis, EGFR signaling, G-protein signaling, & Wnt signaling (in DHF versus ND), Ubiquitin proteasome signaling, nicotine degradation, Wnt signaling, circadian clock, cell cycle, & p53 pathway (in DF versus ND), and Apoptosis, TLR, IGF, p53, blood coagulation, & metabolic pathways (**Figures 2A, B** and **Table 1**).

**Table 1:** Condition-specific pathways: (A) Common DEGs & pathways between DH *vs* DHF and DHF *vs* ND (B) DF—DHF-specific (1 pathway), and (C) DF—ND-specific (11 pathways), and (C) DHF—ND-specific (50 pathways).

Common DEGs	Common pathways	DHF—ND-specific
IGF1	Arginine and proline metabolism	04350 TGF-beta signaling pathway
IFT122	mTOR signaling pathway	00480 Glutathione metabolism
PDLIM7	ABC transporters	00983 Drug metabolism - other enzymes
GJA9-MYCBP	Oocyte meiosis	05010 Alzheimer's disease
C10orf67	Cytosolic DNA-sensing pathway	00040 Pentose and glucuronate interconversions
MRAP2	RIG-I-like receptor signaling pathway	00020 Citrate cycle (TCA cycle)
IRF3	Apoptosis	00670 One carbon pool by folate
TMEM74	Focal adhesion	02020 Two-component system
GS1-103B18.1	Cell cycle - yeast	00140 Steroid hormone biosynthesis
NLGN2	Toll-like receptor signaling pathway	00460 Cyanoamino acid metabolism
MORC3		00600 Sphingolipid metabolism
GJA9	DF—DHF-specific	03050 Proteasome
PAQR3	Complement and coagulation cascades	04130 SNARE interactions in vesicular transport
C7orf49		00590 Arachidonic acid metabolism
FGF4	DF—ND-specific	04940 Type I diabetes mellitus
SIK2	Renin-angiotensin system	00071 Fatty acid metabolism
CYCSP44	Glycosphingolipid biosynthesis - lacto and neolacto series	04270 Vascular smooth muscle contraction
CSMD1	Glycosaminoglycan biosynthesis - chondroitin sulfate	04950 Maturity onset diabetes of the young
SLC7A8	Glycosylphosphatidylinositol(GPI)-anchor biosynthesis	05416 Viral myocarditis
INSIG2	Phototransduction	04330 Notch signaling pathway
ATP11B	Glycosaminoglycan biosynthesis - keratan sulfate	04630 Jak-STAT signaling pathway
MBNL1	Amino sugar and nucleotide sugar metabolism	00625 Tetrachloroethene degradation
DNPEP	Basal cell carcinoma	04020 Calcium signaling pathway
F13B	Lysosome	00361 gamma-Hexachlorocyclohexane degradation
NCAPD2	N-Glycan biosynthesis	00591 Linoleic acid metabolism
TNFRSF10B	Streptomycin biosynthesis	00230 Purine metabolism
HSH2D		00830 Retinol metabolism
LOC101927870		00363 Bisphenol A degradation
REL		00290 Valine leucine and isoleucine biosynthesis
GORASP2		04520 Adherens junction
MYCBP		00270 Cysteine and methionine metabolism
DUSP16		00770 Pantothenate and CoA biosynthesis
CCDC7		00650 Butanoate metabolism
PYCRL		04920 Adipocytokine signaling pathway
LOC653739		05140 Leishmaniasis
ABCA13		04640 Hematopoietic cell lineage
		00260 Glycine serine and threonine metabolism
		00430 Taurine and hypotaurine metabolism
		03020 RNA polymerase
		00620 Pyruvate metabolism
		00970 Aminoacyl-tRNA biosynthesis
		04621 NOD-like receptor signaling pathway
		03030 DNA replication
		00240 Pyrimidine metabolism
		05414 Dilated cardiomyopathy
		05142 Chagas disease
		05310 Asthma
		05222 Small cell lung cancer
		00120 Primary bile acid biosynthesis
		03022 Basal transcription factors

### Immune System and the Associated Pathways Are Major Target for Dengue Virus

**Figure 3:**
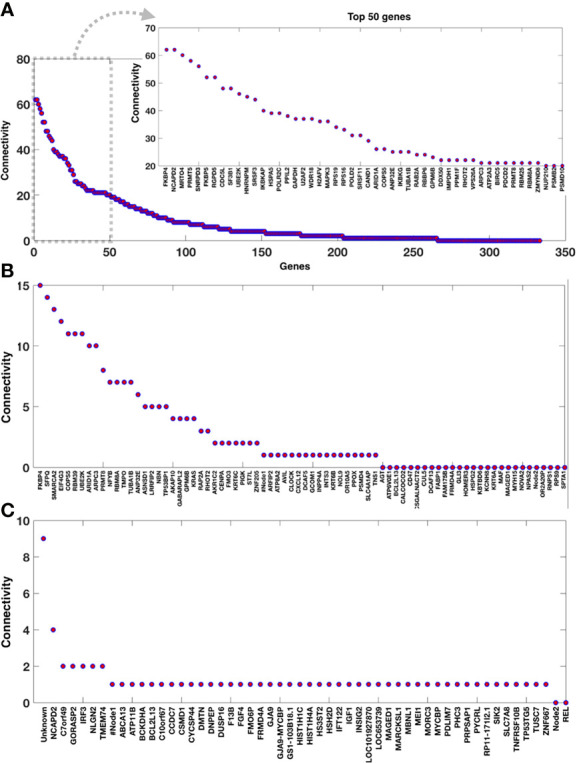
Connectivity of the genes within the DEGs networks. Genes connectivity in the DEG networks **(A)** DHF *versus* ND, **(B)** DF *versus* ND, and **(C)** DF *versus* DHF.

We see a variety of altered pathways based on the inferred pathways that are either part of the immune system or theoretically control/contribute to normal immune system function. As a consequence of dengue infection, their pathway constituents (genes) are predominately present among the DEGs (**Figure 3A–C** and **Table 1**). We have explored the significance of these genes by analyzing the DEGs networks (**Figures 1F–H**) and presented the top-ranked genes based on their connectivity within the specific DEGs network.

### Dengue Infection Shares Critical Biological Pathways With Pneumonia

**Figure 4:**
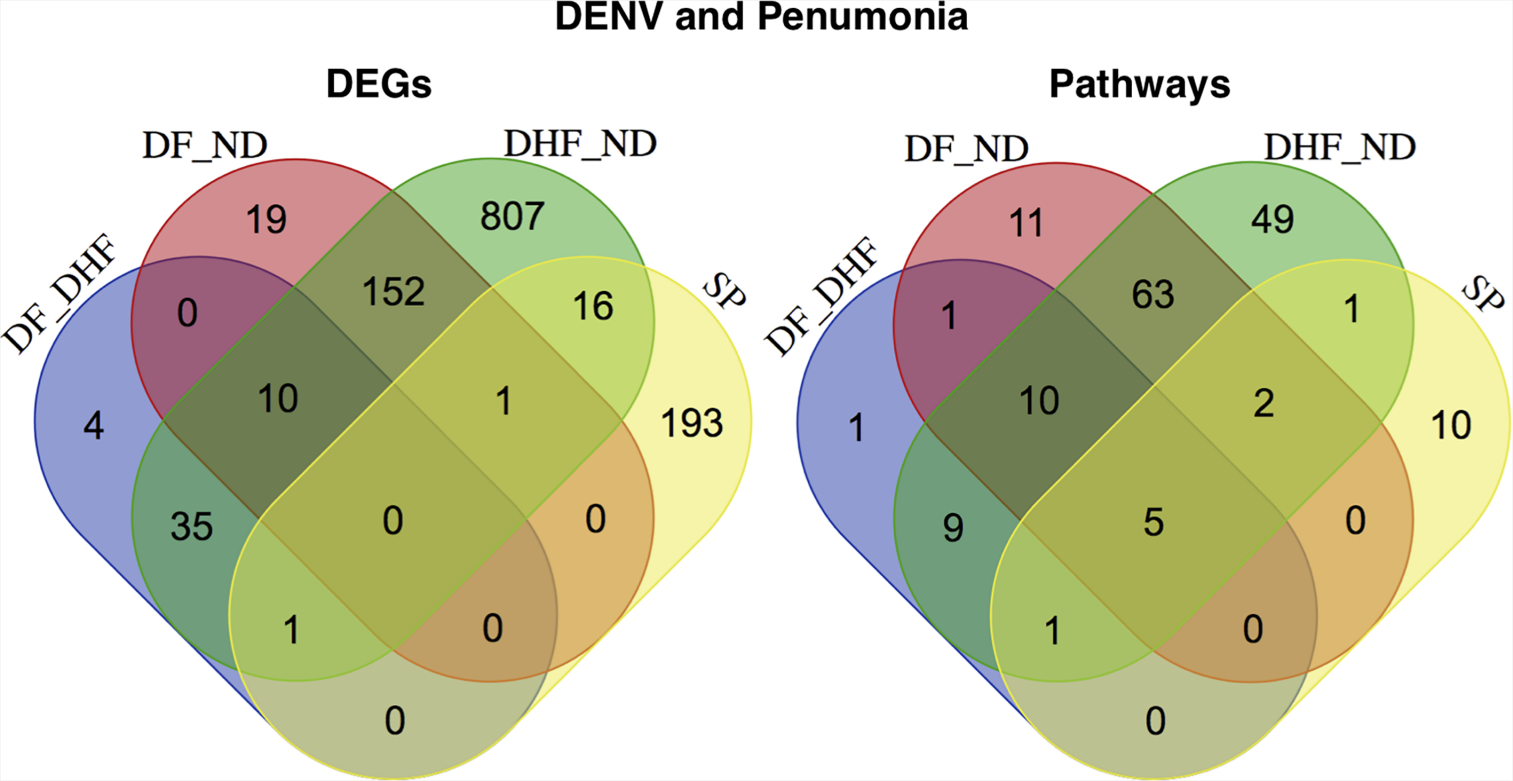
Linkage between the DENV and Pneumoniae. Comparative study of expression patterns and the functions between DENV and SP.

An infectious disease occurs when the immune system of the host is weakened or when the infectious agent overwhelms the immune system and thus the major focus of the study was to deeply unravel the commonly linked immunological pathways and the components. Finally, we have investigated the linkage of dengue infection with pneumonia for which we have compared the DEGs of dengue infected patients with the pneumonia patients DEGs and similarly the compared the enriched pathways. Here, we observe that there are only 16 DEGs (LOC101060835, MBNL2, HLA-DRB4, HLA-DQB1, CXCL8, FKBP5, TRIB1, GAP43, SLC19A1, MAPK1IP1L, CXCL2, KCNV1, HLA-DRB1, CLEC5A, LOC100996809, and HLA-DRB3) which are common in two of the cases (DHF—ND and SP), PDLIM7 DEG appears common between DF–DHF, DHF–ND, and SP, and GPM6B is commonly differentially expressed between DF–ND, DHF–ND, and SP while there are comparatively higher numbers of commonly enriched pathways. Cell adhesion molecules (CAMs), MAPK signaling pathway, natural killer cell mediated cytotoxicity, regulation of actin cytoskeleton, and cytokine-cytokine receptor interaction are commonly enriched in all the three cases of DENV and SP, focal adhesion was enriched between DF—DHF, DHF—ND, and SP, and antigen processing and presentation and Leukocyte transendothelial migration were enriched in DF—ND, DHF—ND, and SP (**Figure 4**). The complete list of DEGs and enriched pathways were supplied as supplementary data (**Table S1** and **S2**).

So, we could say that in terms of gene expression there is less commonality while in terms of the functions they are more common which also means that the commonly enriched pathways also have different sources of functional alteration than the dengue infection or vice-versa. Moreover, we have also explored the the commonly enriched pathways and the shared DEGs by representing the network of pathways and genes by using KEGG pathway database where the phagosome, hematopoetic cell lineage, and cell adhesion molecules are interconnected *via* the HLA-DRB1, HLA-DRB 3, HLA-DRB4, and HLA-DQB1 DEGs while CXCL2 and CXCL8 control cytokine-cytokine interaction, TNF signaling, and NF-kB signaling pathways, and PKBP5 controls estrogen signaling.

## Discussion

Dengue virus is among the leading infectious diseases which are a potential risk among the developing countries (Seema and Jain, 2005) and due to the nature of its devastating impact, it is of potential interest to understand alterations at multiple levels such as gene expression, genetic aberrations, etc. However, there still exists a great challenge to understand the molecular basis of pathogenesis of the different manifestations of dengue virus infections in humans{Halstead:1988iy, Martina:2009bj, Chen:1997km, Datan:2016cw, Bardina:2017dd, Clarke:2002bp}. In the previous study the focus has been towards gene expression profiling (Ubol et al., 2008; Sim and Hibberd, 2016) in dengue infection but, differently from the previous study, the innate immune mechanisms provide the host with the time needed to maximally induce the more slowly developing adaptive immunity. Here, we have mainly presented the gene expression profiling and its potential impact on the signaling pathways. For this purpose, we have not only analyzed the DEGs and inferred pathways but also analyzed the networks of DEGs (**Figures 1F–H**).

As we have mentioned the top 30 genes (up and down regulated) and analyzed the inferred pathways which lead to the critical pathways such as T-cell activation, Angiogenesis, EGFR signaling, G-protein signaling, & Wnt signaling (in DHF versus ND), Ubiquitin proteasome signaling, nicotine degradation, Wnt signaling, circadian clock, cell cycle, & p53 pathway (in DF versus ND), and Apoptosis, TLR, IGF, p53, blood coagulation, and metabolic pathways. Based on this, we conclude that there are some pathways which are exclusively affected for DHF and DF.

In the past few decades a large amount of research on dengue has resulted in a host of literature, which strongly suggests that the pathogenesis of DHF and DSS (dengue shock syndrome) involves viral virulence factors and detrimental host responses (Halstead, 1988; Martina et al., 2009; Nascimento et al., 2009; Costa et al., 2013; Mackow, 2014; Screaton et al., 2015; Thomas, 2015; Sim and Hibberd, 2016; Chatchen et al., 2017). Most importantly the DHF-specific top ranked pathways are T-cell activation which is one of most important part of adaptive immune system. There are other critical pathways specific to DF and DHF which will be helpful to proceed in the direction of precise and improved accuracy diagnosis. A personalized approach to the study of dengue pathogenesis will elucidate the basis of individual risk for development of DHF and DSS as well as identifying the genetic and environmental bases for differences in risk for development of severe disease. So, if we look from future and diagnostic perspectives it is of potential significance and promising for biomarker development and improved diagnostic approach (Eckhardt et al., 2020).

In the previous work, we have exclusively explored (Warsi et al. 2020), but more works have focused on gene expression profiling after DENV infection while different from these works (Ubol et al., 2008; Nascimento et al., 2009; Rothman, 2011; Sun et al., 2013; Datan et al., 2016; Sim and Hibberd, 2016; Simon-Lorière et al., 2017), we have compared the two datasets in terms of expression patterns and the altered functions. We were interested in learning more about the potential connection between DENV and SP infection in humans since the majority of infectious diseases are linked to the immune system, and our aim was to learn more about the infection’s effect on the immune system. An infectious disease occurs when the immune system of the host is weakened or when the infectious agent overwhelms the immune system and thus the major focus of the study was to deeply unravel the commonly linked immunological pathways and the components. Finally, we have investigated the linkage of dengue infection with pneumia for which we have compared the DEGs of dengue infected patients with the pneumia patients DEGs and similarly the compared the enriched pathways. These works will help to improve understanding and diagnosis for therapeutic purposes and also to explore the comparative understanding for furture purpose. Such works could also be utilized for the purpose of modeling and simulation of the specific pathways to understand the dynamics of the pathways components and will help to explore the pathways crosstalk, interactions, and the loops (Mobashir et al., 2012; Mobashir, 2013; Mobashir, 2014).

## Conclusion

Dengue fever is a life-threatening illness that affects approximately 2.5 billion people in dengue-endemic areas. Dengue viruses have a single-stranded positive RNA genome. By analysing gene expression results, we hoped to learn more about the pathways caused by dengue infection. Our findings suggest that in the case of dengue infection, possible dengue virus targets are associated with the immune system or mechanisms associated with it, as well as their components. These results have mechanistic implications for how the innate immune system modulates dengue virus transmission and pathogenesis. We also found that DENV and SP infection have the potential to affect immune signalling pathways through their components, and that there are many essential pathways that are shared between the two cases.

## Methods

In this study, we used GEO to obtain gene expression datasets (Nascimento et al., 2009) (GSE18090) for mild, dengue fever, and dengue hemorrhagic fever, as well as a dataset (Ramilo et al., 2007) (GSE6269) for pneumoniae. Dengue datasets were collected and analyzed from the beginning to the end, from simple steps to review and interpretation. The Affymetrix Human Genome U133 Plus 2.0 and U133A arrays were used to construct these gene expression profiling datasets. The selected DENV and SP datasets are defined in simple terms.

The samples are blood samples from 26 patients from a Brazilian cohort, who were divided into three classes, DHF, DF, and ND, as follows: where 18 were confirmed dengue 3, genotype III cases, ten of which were diagnosed as dengue hemorrhagic fever (DHF) and eight as classical dengue fever (DF), as well as eight control samples (ND) from febrile patients who were confirmed to be dengue-free. At the time the samples used in the functional genomic characterization were obtained, none of the DHF patients had any signs or symptoms of vasculopathy. These patients had been sick for around 5 days at the time of collection, and the absence of fever was confirmed two to three days later. To avoid major variations in patient age, gender, dengue infection history, and days of symptoms, the samples from the DF, DHF, and ND patients were matched (Nascimento et al., 2009).

Each infectious agent is made up of a different set of pathogen-associated molecular patterns that interact with immune cells’ pattern-recognition receptors. As a result, they have hypothesized that the blood immune cells of people with various infections may have distinct transcriptional signatures. For the peripheral blood samples from pediatric patients with acute infections, gene expression profiles were collected (Ramilo et al., 2007).

In short the basic steps involved for the entire study are raw file processing, intensity calculation and normalization. For normalization (Girke, 2000; Ideker et al., 2000; Quackenbush, 2002; Simon, 2008), GCRMA (Salomonis et al., 2007; Bild et al., 2009; Reimers, 2010; Chen et al., 2014), RMA, and EB are the most commonly used approaches. Here, we have used EB for raw intensity normalization. After normalization, we proceeded to our goal which was to understand the gene expression patterns (Lapointe et al., 2004; Subramanian et al., 2005) and their inferred functions (Subramanian et al., 2005; Mi et al., 2016). For the pneumoniae dataset, GEO2R (Davis and Meltzer, 2007; Li et al., 2008; Reis et al., 2011) was utilized for DEGs prediction.

For differential gene expression prediction and statistical analysis, MATLAB functions (e.g., mattest) were used. Mattest performed a two-sample t-test to evaluate differential expression of genes from two experimental conditions or phenotypes and it processes a matrix of gene expression values where each row corresponds to a gene and each column corresponds to a replicate. Further, we proceeded to find p-value corrections and FDR (false discovery rate). The basis of DEGs selection were fold changes and the p-values and the limit for fold changes were +/- 2.0 (upregulated and downregulated genes) and the p-values were less than 0.05. For pathway analysis, we used panther database (Mi et al., 2016) and have our own code designed for pathway and network analysis. The principle of enriched pathways remains similar to DAVID database where we have a list of background and target genes. The complete approach for this study has been presented in **Figure 1A** which was also applied for the previous study (Kamal et al., 2020; Kumar et al., 2020; Warsi et al. 2020; Eldakhakhny et al., 2021).

For generating the DEGs network, FunCoup2.0 (Alexeyenko and Sonnhammer, 2009) was used for all the networks throughout the work where the intra-connectivity between the list of DEGs was fetched and cytoscape was used for network visualization. In most of the steps of this work, we used our own coding and in the calculations MATLAB was used. In general, the principle of FunCoup is that it predicts four different classes of functional coupling or associations such as protein complexes, protein-protein physical interactions, metabolic, and signaling pathways (Alexeyenko and Sonnhammer, 2009).

## Data Availability Statement

The original contributions presented in the study are included in the article/**Supplementary Material**. Further inquiries can be directed to the corresponding authors.

## Author Contributions

Conceptualization, LB, SS, TA, MK, SP, and EA. methodology, LB, SS, MM, MK, and EA; software, EA; validation, LB, TA, and EA; formal analysis, LB, SS, MM, MK, EA; investigation, LB, SS, MK, and EA; resources, SS, MK, and EA; data curation, SS, MR, AF, MM, and EA; writing—original draft preparation, LB, SS, MM, MK, and EA; writing—review and editing, LB, SS, TA, MM, MK, SP, MR, AF, and EA; visualization, LB, SS, MR, AF, and EA; supervision, MK, and EA; project administration, EA; funding acquisition, MK and EA. All authors have read and agreed to the published version of the submitted version.

## Funding

This research was funded by Deanship of Scientific Research (DSR) at King Abdulaziz University, Jeddah, Saudi Arabia, grant number FP-5-42 and The APC was funded by FP-5-42.

## Conflict of Interest

The authors declare that the research was conducted in the absence of any commercial or financial relationships that could be construed as a potential conflict of interest.

## Publisher’s Note

All claims expressed in this article are solely those of the authors and do not necessarily represent those of their affiliated organizations, or those of the publisher, the editors and the reviewers. Any product that may be evaluated in this article, or claim that may be made by its manufacturer, is not guaranteed or endorsed by the publisher.
