# Supplementary material for: Gene Expression Profiling of Early Acute Febrile Stage of Dengue Infection and Its Comparative Analysis With Streptococcus pneumoniae Infection
Source: Front Cell Infect Microbiol. 2021 Oct 28;11:707905. doi: 10.3389/fcimb.2021.707905 (PMC8581568; doi:10.3389/fcimb.2021.707905)
Supplement: Supplementary file 1 [file Table_1.docx]

**Table S1.** Specific and common DEGs for DENV and Pneumonia

| **.Names** | **total** | **elements** |
| --- | --- | --- |
| DENV Pneumonia | 18 | LOC101060835 MBNL2 HLA-DRB4 PDLIM7 HLA-DQB1 CXCL8 FKBP5 TRIB1 GAP43 SLC19A1 MAPK1IP1L CXCL2 KCNV1 HLA-DRB1 CLEC5A LOC100996809 GPM6B HLA-DRB3 |
| DENV | 1025 | MSRB1 RAB2A TMEM239 PTGER3 RP1-63G5.8 ZFP92 C10orf25 NARR PDHA1 LOC101928098 NBN EPR-1 BID LOC400622 FGL2 CEP70 MACF1 ADAMTS18 TAF5L IGF1 TGFB2 LDB3 IFT122 PAK1 NBPF12 DCAF5 C1R APOBEC4 DAZAP1 FAM134B MEX3B ZNF385D NID1 RP11-654A16.3 GYS1 SLC2A5 PACSIN2 SLC33A1 SLC25A37 LOC102723809 AX747250 TOPBP1 HOMER3 AC005306.3 TCL6 RPL34-AS1 TRMT61B MET GABARAP INIP ING3 AK7 ARL1 SYNPO2 SSH2 RNF41 NGF MRPL38 UGT1A1 RRP7A ANP32E HERC2P2 TPP2 RNASE6 LINC00698 GJA1 SAMD3 POLR3K LAX1 PCDHAC1 B4GALNT1 AGAP11 SFPQ IRX5 PTPN22 SLC12A4 GGT2 EXT2 TCEB3CL CACTIN SMIM3 LOC102724884 LAMP1 EIF4G3 CAND1 STX2 RP1-263J7.1 ACSL3 TMEM18 VPS26A RMDN2 LYSMD4 TBL3 FLJ32742 GJA9-MYCBP ABHD4 KIAA1430 GRK5 HES1 SNRNP70 LINC01097 SLC35F6 LINC01138 CSGALNACT2 TMCO3 LINC01209 TAF5 SRRM4 C10orf67 BIRC5 LOC643711 CARNS1 SLC1A7 ASB8 KRTAP5-2 NOA1 NKD1 DCBLD2 TAPBPL RBM33 BTG1 KIAA1715 HTR7 OR5H1 ASB10 MEIS3P1 KB-1568E2.1 A2ML1 C14orf28 ANKRD20A3 LGALS1 DOCK8 PLEKHB1 LINC01019 EMC1 PTH2 KIAA1211L HTR7P1 PTK2 KLHL36 MXD4 TMC2 KIAA0100 FNIP1 LOC101928107 SRSF11 PARVA PSMB6 CAMTA1 MRAP2 PCDHB8 NOVA2 LOC101929225 TMEM151B LINC00515 ZNF41 RP11-416I2.1 KBTBD6 C9orf16 CD6 SSX7 CELF1 ZNF836 SEC23IP EDNRA EDRF1 F9 MPPED1 LSM4 B4GALT1 CNGB3 NTNG2 PTMS LDHB IRF3 POM121L12 NBPF8 CD40 TACR1 FAM135B DYNC1LI2 E2F1 PRRC2B EFCAB7 ZMYND8 DOHH PHLDA1 LINC01207 RAB11FIP2 CFH INO80D PRMT2 SETMAR NCAM1 LOC283352 METRN AGT MYOT KRAS ATP7A RGPD5 CDX2 HIRA MERTK ASNSD1 ARMC8 ZNF70 CXorf67 RARB ZNF227 MCOLN2 HTR3C NGDN CDH8 RPUSD3 C15orf62 ZNF639 AKR1C2 MYO10 ARPC3 TMEM74 TRPV5 ZNF527 RASEF POMZP3 FKBP4 GIPR VWA1 OR2A9P AKR7A3 CSTF2T AHCYL1 RP11-171I2.1 GS1-103B18.1 DNAAF1 RAB21 LOC100130111 TUBA1B NLGN2 LOC101929741 C10orf76 HK3 ALPP RBPMS NAA16 ATP6V1C1 ATP10A CC2D1A MLIP ZBTB43 EGFL7 NFE2L2 FAM195A TGM5 YWHAZ ACAD11 D21S2088E RBBP6 SKAP2 IL17A PFKL TMPO IDS ZNF667 LEPR DISC1 LOC101928989 POLD2 PAX8-AS1 KCNB2 NPHP3-ACAD11 SPINT3 RIC3 USP13 ANKRD20A1 C17orf75 SLC43A3 ZNF709 NBPF20 EFNA2 ZNF20 LOC554174 LEPROT EPS8L1 KSR1 RRP7B CENPJ KCTD10 ADRB1 IGHM LOC101929289 SMPD1 MORC3 SVIL OAS1 TH MYOF MFSD2A C21orf49 MRTO4 LUC7L PMS2CL LOC401320 AKAP10 ZIC1 DEFB124 OR2H1 EFCAB14 IKBKG ARL10 GJA9 RP11-65D24.2 PAQR3 CTD-2313J17.5 OR6W1P SPON1 LRRC42 SENP6 MYCNOS ASTN2 FAM9B AVIL PRMT8 SNX13 PSMB2 LINC01225 VIM PSKH2 IFT43 RAB9B NEU3 MYOZ2 DCT FBF1 SERPINB13 UGT1A8 LRBA SERP1 SCD PEX13 PPOX TMPRSS4 SLC39A9 C7orf49 DENND4B PLEKHM3 TPCN2 SPP1 TTC39C TSSK3 C14orf159 EIF2B2 ANKRD20A5P MPP2 NOP16 ZSWIM8 EMC9 PEX26 LAMTOR5 GGTLC2 ASCC2 SLC44A1 WBP11 SHPRH ARFGEF2 FGF4 MAP1A ALPPL2 GPRC5A FIS1 LRPPRC COL19A1 BMP15 ITGA6 KCNG2 SALL2 SLC16A2 LOC100507516 NOTCH2 ANKRD54 KDM6B TUSC7 TTC28-AS1 CCDC79 HIST1H3G LST1 TAF1D SFRP4 ADAMTS20 CENPA LOC101928605 CCDC147 TYMS FMOD DCAF8L2 CLCA2 SIK2 PPIB LINC01191 CYP2A13 TNS1 RRBP1 CALCOCO2 ITK MED6 CDK16 LOC100506538 BSDC1 THEM4 GNG2 ZNF677 CYCSP44 UBE2K XPOT KY TSPY26P OR1Q1 CSMD1 FUT8-AS1 SOCS4 HPGD LINC00896 DNAJC18 PRPSAP1 ASB16 PITPNM2 GLI3 GRAMD4 TP53BP1 CEP68 GAST AK2 KRT34 SEPT7P9 SCART1 FERD3L FRMD4A RPS4Y1 ARSA C1QTNF9 BIRC2 ILF3 NTRK3 H2AFX PIGK BCL2L13 DACT3-AS1 ANKRD20A12P ELMOD2 RQCD1 PIANP ARFIP2 GP2 TXLNA RAB31 DSERG1 ZFAND6 IGLVIVOR22-1 KIAA0087 FAM173A LOC101928274 CTNNA3 RDH11 AK055055 SRSF3 DLGAP1-AS2 ADPRHL1 KRT6A PRR14 MRPL37 SLC7A8 NPHP3 LINC01165 MMP1 SAMD5 TFAP2A-AS1 HYAL2 POLR2M RBPJL IKBKAP MIR4745 ERVW-1 GRAMD1A CTDP1 WNT16 USP37 PTBP1 DDX50 ANAPC10 BC042590 SLAMF6 ATP2A3 PDCD2 LIMK2 APOF RBSG3 TMPRSS15 INTS3 TOR1A GADD45B RP11-744D14.2 ZP3 NUP210 C21orf15 IDH3A LOC100130429 SLC25A30 YWHAB LOC101927121 RP11-752D24.2 DNAJC6 SNAP29 CLOCK SMR3A YBX1 GPR155 PNISR ZNF548 CCDC149 SSX3 LOC729224 NPAS2 CLUHP3 LETM2 WBP1L ZDHHC18 PDCD6 GGT1 CCSER2 SULT1E1 RGPD4 ADRBK1 OGT ST3GAL2 PGAM5 RNF207 MUC19 FOXP2 INSIG2 ATP11B EXT1 PTPRS SHCBP1 NPHP4 U2AF2 DOCK9 CCS LOC100996506 ZDHHC1 COMMD4 ZNF580 CENPE IFNA7 C14orf183 CSNK1E PTPRD SASH1 MYH15 KLHDC7A CDC14B NSF LOC100132686 ANKRD20A2 SCARA5 HRH3 MAOB PLCH1 FLCN SMURF1 UGT1A9 COPS5 ESAM OR2A1-AS1 LINC00950 OPTN STX17 ZNF619 BCL2L11 SERINC5 ELP5 ST7L CTNNA1 ELFN2 SCAMP4 GDAP2 SP110 C1QTNF9B RPL7P27 SH3BP2 PRMT5 PRPH2 KCNH8 CUL5 MSH4 RP11-251G23.5 OPN5 RP3-496C20.1 ID4 UGGT2 SPRY1 FABP1 NFIB FMO3 MDM2 TAP2 TCEB3C FBXO9 HSPA9 FMO6P LOC101929505 IRF2BP1 GPR161 LOC102724323 NBPF10 TSPO SNX9 MBNL1 LINC01428 KRT6C DNPEP PURA GPR144 ABI1 ARHGEF15 RP11-421E14.2 CDX4 SPG21 TMPRSS3 ANKFY1 TBC1D1 HSPA5 DENND3 OR2A20P CD28 SMARCA4 FIP1L1 ENSA ZNF449 GCOM1 DTYMK PSD4 F13B PDZD8 RGS11 RP11-116D17.1 HEXDC CXCL12 SH3D21 SLC39A13 NTN1 HNRNPM IL36A RP11-401P9.4 TCEB3CL2 EEF1E1 FBXL21 AC007349.5 HM13 HELB PHACTR2 SLC4A1AP APOLD1 EHD4 CELA2B GPR50 PPIL2 NR5A2 FEN1 RIPK1 FOXI1 BCKDHA DNAAF2 SF3B1 EFNB2 ICAM1 MTHFS PLEKHH1 ABAT PCM1 LOC101928717 LHX6 MAPK3 FAM175B LOC389906 OR5J2 ABCF3 PCDH10 CELSR1 MKL2 TMEM38B CSN1S2AP C16orf72 LRRC7 NCAPD2 SPTA1 CCDC136 ANK1 FAM90A1 SHB RAD50 HERC2P9 GATA5 RHOT2 ERO1LB ABCB4 PSMD10 ARPC1B C2CD2L IGHG1 EXTL3 SETDB2 HRH1 TNFRSF10B TMEM97 TBX19 PAX8 HSH2D RPS19 ZBTB10 DCAF13 STRA6 TCEA1 BTN2A3P C16orf46 TRIM52-AS1 C8orf17 IST1 LOC100507600 RAB34 SIGLEC10 OR5I1 ARHGAP5 WFDC11 ZFP2 JMJD1C-AS1 PSMD4 CACNB2 C6orf195 BC027448 REPS1 TNRC6C MAP3K4 LINC01208 LIN9 LOC101930165 CEBPA-AS1 SPATA6L ZNF99 ZNF205 SDC3 LOC728613 RUSC2 PRMT3 LOC101928327 H2AFV LOC101927870 LOC101930171 LYNX1 ZNF625-ZNF20 KRT19 GABRB3 GNB5 GOSR2 SP3 LOC101930400 RAP2A RNPS1 LOC101930324 MAP1B POU2F3 LINC00955 DNAH8 MYO7A PANK2 ADAM8 SLC9C2 CCDC69 OFCC1 REL EWSR1 BICD2 NOL9 C14orf119 LPAR6 ZNF560 TM4SF1 PLEKHH3 SMG7 RPL35A PSORS1C2 DGCR12 LRRFIP2 PEX10 HLA-DMA LOC100653049 ZNF93 RP6-149D17.1 TAB3 OR5P2 GGTLC1 LRRN4CL AKR1C1 MARCKSL1 C2orf15 DYNC2LI1 ZNF575 ANGPTL2 RP11-209D14.2 TTC6 LOC101928844 NEDD9 HS3ST2 TIRAP HEY2 GORASP2 PSMD13 CXCL5 MGC4859 RBM8A ZNF660 ITGA9 LOC149373 S1PR5 MYCBP MEI1 MGC15885 TYRO3P IDUA CCND2 LCP1 CFLAR PITRM1 MAF UGP2 TRAK1 EIF4A2 RABGAP1L C16orf95 CD47 LAP3 PPM1F SNRPD3 IMPDH1 NFYB WDR18 FDXR RBM39 ANKRD20A4 POLR2C LOC101929858 MIR4800 KLC2 ELTD1 ZNF28 FBXO38 LINC00612 KIAA0430 ATRNL1 SLFN5 SLC16A8 MARS QRSL1 PREX1 RGPD8 ANKRD20A8P PPIE VOPP1 SNX24 SMARCA2 LOC100505716 2-Sep INPP4A RGPD3 C21orf90 ATP8A2 AFTPH CALD1 BSND RP11-288H12.4 ERICH1 FAM9C C1orf213 LARP4 DUSP16 IL9 ZNF503-AS2 IL11 NBPF9 DTWD1 RFX4 KRT6B SOBP LINC01234 PKP3 LOC100130938 WWP2 CTSH DNAL1 FBXL18 BECN1 SORBS3 LINC01426 IFT57 FREM1 DNTT PAGR1 GRK7 CDC5L OR10A5 SLC9B1 NDUFA10 NBPF14 TDRD1 LOC101060464 KHDRBS1 MON1A GAPDH FAM131B APC PMP22 LOC101927792 DBR1 C14orf2 COMMD6 FUT2 DENND6B OR3A2 RSL1D1 LOC101928481 PARD3B HIST1H1C RBM25 HGSNAT KIAA0754 MEX3C KPNA6 HIST1H4A SERPINA3 MAGED1 ARMCX4 CCDC7 STK38 MTCH2 LOC101927003 GAB1 PNN LOC101928476 C15orf37 MRPL30 GABARAPL2 LOC441528 SAMD4B GLP2R SETD4 VSIG10 CDKN3 LOC101927126 BMP5 ZMYM6 GABRA3 RPS16 LOC101929373 CSNK2A1 FGD6 HSPG2 RPS9 ATP6V0E1 NDFIP1 HEATR2 RP11-384P7.7 PYCRL AAK1 SULT1C4 LINC00167 SSX5 RP11-79P5.2 TMF1 AKT3 SFRP1 CAAP1 SCML4 TMED4 GGA2 TRIB2 UGT1A3 MIR6758 EVL UGT1A5 ATP8B1 PKD2L2 ARID1A PRODH2 RGPD6 GINS2 ST20-MTHFS GATA4 LOC653739 VGLL4 MS4A14 RPS6KA3 SNORD8 BRWD1 LOC284513 RP11-819C21.1 UQCRC2 NBPF15 PATL1 8-Sep ABCA13 TMEM63A SAFB AVP LOC101928623 CCDC13 PMS2 SENP5 RPL39L TOP1 DCAF8 PCP2 RP11-37C7.3 SHISA5 SLC39A6 FZR1 TP53TG5 |
| Pneumonia | 193 | LOC149684 LMAN1 LILRA4 DAZ3 AGFG1 GNAL KIR2DL2 S100P CEACAM6 HLA-DRB5 GK CEP135 CLN8 ANXA3 ELANE DEFA4 POU1F1 CEACAM8 EPB42 ORM1 SLC2A3 C8orf4 SBNO2 SLC6A6 NME8 PDE4C RNASE2 TKTL1 PLD1 LOC401317 CHI3L1 EPHA4 DSC2 SPRY2 KIR3DL2 CYP4F3 HBG2 PCSK6 EGR1 FAR2 THBD HP PDE9A TNFAIP6 STEAP4 MAPK14 SERPINB2 HPR PXN TLL2 ADGRL4 RAPGEF1 ANO1 EGR2 NCR3 HIST1H2BB MPO CPD DEFA1B PGLYRP1 RHAG CD177 GM2A LOC100293211 SPDL1 ARG1 EIF5A HBD SLPI FCAR CCL4 DAPK2 PRF1 EREG CAMP SNCA EIF3F KIR3DL1 ARHGAP26 HBQ1 HIPK3 FAM30A WSB1 DAZ1 MME TMEM259 SOCS3 PLK4 LOC729966 CHIT1 AZU1 THAP3 KIR2DL1 CRISP3 XCL2 RAB11B CREB5 UBASH3A HLA-DRB6 FKBP8 ADGRG1 SEC23A XCL1 DEFA3 SLCO4A1 MYBL1 COL9A3 MGAM ASGR2 HNRNPH1 GZMH TMEM158 FOXO3B SLC2A14 BRCA2 CD24 ITGA2B MYOM2 NAMPT MMP8 DUSP6 FAM198B TCN1 CA4 CLIC3 LRP1 OLFM4 SPI1 RNASE3 PADI4 SNAP23 SURF1 CYP4F2 IL1R2 APOH CDH1 FOS RETN DEFA1 KIR2DS2 MED1 CLU GLUL TACSTD2 GYPB PRTN3 THBS1 GZMB FCGR3B LTF NPR2 TMEM144 LRRN3 E2F2 TCF3 TMOD1 MYL9 JAK3 PATJ FAXDC2 PTPRJ MXD1 ORM2 ZNF135 FOSB ITGB3 MARCO DAZ2 LCN2 ALAS2 AKR1C3 ELL2 CNIH3 FOXO3 BPI CA1 KCNJ15 NRG1 LOC100507472 DDX3X PLAUR TMOD3 IL1R1 SELENBP1 FOSL1 TTC38 AHSP TUBB1 CYP1B1 DAZ4 HBG1 CCDC88A MMP9 |
