# Supplementary material for: Gene Expression Profiling of Early Acute Febrile Stage of Dengue Infection and Its Comparative Analysis With Streptococcus pneumoniae Infection
Source: Front Cell Infect Microbiol. 2021 Oct 28;11:707905. doi: 10.3389/fcimb.2021.707905 (PMC8581568; doi:10.3389/fcimb.2021.707905)
Supplement: Supplementary file 2 [file Table_2.docx]

**Table S2**: Enriched pathways specific and common between the two infections.

| **Names** | **total** | **elements** |
| --- | --- | --- |
| DENV Pneumonia | 17 | KEGG_04514_Cell_adhesion_molecules_(CAMs) KEGG_04151_PI3K-Akt_signaling_pathway_-_Homo_sapiens_(human) KEGG_04024_cAMP_signaling_pathway_-_Homo_sapiens_(human) KEGG_04380_Osteoclast_differentiation KEGG_04668_TNF_signaling_pathway_-_Homo_sapiens_(human) KEGG_04640_Hematopoietic_cell_lineage KEGG_04650_Natural_killer_cell_mediated_cytotoxicity KEGG_04015_Rap1_signaling_pathway_-_Homo_sapiens_(human) KEGG_04064_NF-kappa_B_signaling_pathway_-_Homo_sapiens_(human) KEGG_04670_Leukocyte_transendothelial_migration KEGG_04145_Phagosome KEGG_04010_MAPK_signaling_pathway KEGG_04915_Estrogen_signaling_pathway KEGG_04810_Regulation_of_actin_cytoskeleton KEGG_04510_Focal_adhesion KEGG_04917_Prolactin_signaling_pathway KEGG_04060_Cytokine-cytokine_receptor_interaction |
| DENV | 63 | KEGG_04722_Neurotrophin_signaling_pathway KEGG_04910_Insulin_signaling_pathway KEGG_03440_Homologous_recombination KEGG_04071_Sphingolipid_signaling_pathway_-_Homo_sapiens_(human) KEGG_04740_Olfactory_transduction KEGG_03320_PPAR_signaling_pathway KEGG_04110_Cell_cycle KEGG_04350_TGF-beta_signaling_pathway KEGG_00350_Tyrosine_metabolism KEGG_04072_Phospholipase_D_signaling_pathway_-_Homo_sapiens_(human) KEGG_04261_Adrenergic_signaling_in_cardiomyocytes KEGG_04152_AMPK_signaling_pathway_-_Homo_sapiens_(human) KEGG_04144_Endocytosis KEGG_04146_Peroxisome KEGG_04150_mTOR_signaling_pathway KEGG_04660_T_cell_receptor_signaling_pathway KEGG_04666_Fc_gamma_R-mediated_phagocytosis KEGG_04066_HIF-1_signaling_pathway_-_Homo_sapiens_(human) KEGG_04114_Oocyte_meiosis KEGG_04068_FoxO_signaling_pathway_-_Homo_sapiens_(human) KEGG_03015_mRNA_surveillance_pathway KEGG_04371_Apelin_signaling_pathway_-_Homo_sapiens_(human) KEGG_04512_ECM-receptor_interaction KEGG_04919_Thyroid_hormone_signaling_pathway KEGG_04012_ErbB_signaling_pathway KEGG_00240_Pyrimidine_metabolism KEGG_04130_SNARE_interactions_in_vesicular_transport KEGG_03050_Proteasome KEGG_04723_Retrograde_endocannabinoid_signaling KEGG_00450_Selenoamino_acid_metabolism KEGG_04540_Gap_junction KEGG_00340_Histidine_metabolism KEGG_03013_RNA_transport KEGG_04022_cGMP-PKG_signaling_pathway_-_Homo_sapiens_(human) KEGG_04210_Apoptosis KEGG_04340_Hedgehog_signaling_pathway KEGG_04924_Renin_secretion KEGG_04630_Jak-STAT_signaling_pathway KEGG_04550_Signaling_pathways_regulating_pluripotency_of_stem_cells KEGG_04914_Progesterone-mediated_oocyte_maturation KEGG_00500_Starch_and_sucrose_metabolism KEGG_04916_Melanogenesis KEGG_04141_Protein_processing_in_endoplasmic_reticulum KEGG_04020_Calcium_signaling_pathway KEGG_04370_VEGF_signaling_pathway KEGG_04530_Tight_junction KEGG_00052_Galactose_metabolism KEGG_04662_B_cell_receptor_signaling_pathway KEGG_00230_Purine_metabolism KEGG_03040_Spliceosome KEGG_00190_Oxidative_phosphorylation KEGG_04080_Neuroactive_ligand-receptor_interaction KEGG_00380_Tryptophan_metabolism KEGG_04115_p53_signaling_pathway KEGG_03010_Ribosome KEGG_04610_Complement_and_coagulation_cascades KEGG_04310_Wnt_signaling_pathway KEGG_04620_Toll-like_receptor_signaling_pathway KEGG_04392_Hippo_Signaling_Pathway KEGG_04360_Axon_guidance KEGG_04120_Ubiquitin_mediated_proteolysis KEGG_04520_Adherens_junction KEGG_04014_Ras_signaling_pathway_-_Homo_sapiens_(human) |
| Pneumonia | 2 | KEGG_04611_Platelet_activation KEGG_04612_Antigen_processing_and_presentation |
